# A clinical protocol to inhibit the HGF/c-Met pathway for malignant mesothelioma with an intrapleural injection of adenoviruses expressing the NK4 gene

**DOI:** 10.1186/s40064-015-1123-3

**Published:** 2015-07-16

**Authors:** Yuji Tada, Kenzo Hiroshima, Hideaki Shimada, Naoya Morishita, Toshiro Shirakawa, Kunio Matsumoto, Masato Shingyoji, Ikuo Sekine, Koichiro Tatsumi, Masatoshi Tagawa

**Affiliations:** Department of Respirology, Graduate School of Medicine, Chiba University, Chiba, Japan; Department of Pathology, Tokyo Women’s Medical University Yachiyo Medical Center, Yachiyo, Japan; Department of Surgery, School of Medicine, Toho University, Tokyo, Japan; Kobe University Graduate School of Health Science, Kobe, Japan; Divison of Translational Research for Biologics, Department of Internal Medicine, Kobe University Graduate School of Medicine, Kobe, Japan; Division of Urology, Department of Surgery, Kobe University Graduate School of Medicine, Kobe, Japan; Division of Tumor Dynamics and Regulation, Cancer Research Institute, Kanazawa University, Kanazawa, Japan; Division of Respirology, Chiba Cancer Center, Chiba, Japan; Department of Medical Oncology, Faculty of Medicine, University of Tsukuba, Tsukuba, Japan; Division of Pathology and Cell Therapy, Chiba Cancer Center Research Institute, 666-2 Nitona, Chuo-ku, Chiba, 260-8717 Japan; Department of Molecular Biology and Oncology, Graduate School of Medicine, Chiba University, Chiba, Japan

**Keywords:** Mesothelioma, Adenovirus, HGF, c-Met, NK4

## Abstract

**Background:**

The hepatocyte growth factor (HGF)/c-Met signal pathway is up-regulated in human mesothelioma and suppression of the HGF/c-Met signaling with a competitive inhibitor, NK4 homologous to HGF in the structure, produced anti-tumor effects to mesothelioma in a preclinical study. Mesothelioma is highly resistant to a number of chemotherapeutic agents but distant metastasis to extra-thoracic organs is relatively infrequent until the late stage.

**Methods/design:**

We planned to conduct a clinical study of gene therapy with adenoviruses expressing the *NK4* gene (Ad-NK4) to control the local tumor growth. The study is designed to inject Ad-NK4 into the intrapleural cavity with a dose escalation manner from 10^10^ to 10^12^ virus particles per patient and to examine safety and possible clinical benefits. The clinical investigation is a first-in-human trial to use the *NK4* gene and to block the HGF/c-Met pathway with gene medicine. We conducted in vivo animal experiments to examine the safety level as one of the preclinical studies, and showed that Ad DNA administered in the pleural cavity was detected in many parenchymal organs. Biochemical and pathological analyses showed that liver damages were the major adverse effects with little toxicity to other organs. These studies firstly demonstrated biodistribution and transgene expression after an intrapleural injection of Ad vectors in an animal study, which contrasts with an intravenous injection showing relatively rapid clearance of Ad-NK4.

**Discussion:**

The clinical study can also provide information regarding production of NK4 protein and antibody against NK4, and inhibition levels of the HGF/c-Met pathway by detecting dephosphorylation of c-Met in mesothelioma cells. These data will be crucial to judge whether local production of NK4 molecules can be an anti-cancer strategy.

Trial registration: UMIN clinical trials registry, Japan. Register ID: UMIN15771

## Background

Malignant mesothelioma is developed from mesothelium and is associated mostly with occupational and less frequently with non-occupational asbestos exposures (Carbone et al. 2002; Robinson et al. 2005; Porpodis et al. 2013; Røe and Stella 2015). A majority of mesothelioma, about 75% of the cases, is of pleura origin followed by peritoneum. Malignant pleural mesothelioma tends to invade into the vicinity and disturbs functions of vital organs, which results in respiratory and cardiac failure, and spinal cord compression. Distant metastasis to extrathoracic organs is however infrequent until the late stage. The latent period of mesothelioma after asbestos exposure is long, more than 30 years in average, and a medical procedure to prevent the tumor development is currently unknown. Detection of mesothelioma at an early stage is difficult because it is often asymptomatic and the signs and symptoms are not specific to mesothelioma. Moreover, differential diagnosis from other cancerous and non-cancerous diseases needs careful pathological examinations including several kinds of immunohistochemical staining.

Many industrial countries have inhibited asbestos usage, but emerging countries do not have a strict legal regulation. The asbestos consumption in the emerging countries rather increases in line with their economic development and an epidemiological analysis predicts an growing number of the patients in the countries particularly in Asia (Lin et al. 2007). Moreover, mesothelioma is resistant to current therapeutics despite recent multimodal treatments (Carbone et al. 2002; Robinson et al. 2005; Opitz 2014; Kotova et al. 2015). Extrapleural pneumonectomy is one of the standard surgical procedures but it is applicable only to an early-staged case. The recurrence is common even after the radical operation. Mesothelioma is essentially resistant to radiotherapy, which is used primarily for a palliative purpose. Systemic chemotherapy is thereby the primary treatment in most of the cases, and a combination of cisplatin (CDDP) and pemetrexed (PEM) is the first-line chemotherapy regimen over 10 years. Nevertheless, a mean survival rate with the CDDP plus PEM combination is merely 12.1 months (Vogelzang et al. 2003). Different possible chemotherapy regimens have been tested but none of them produced better clinical outcomes than the current first-line chemotherapy.

Biochemical analyses demonstrated aberrant growth signaling with enhanced angiogenesis in mesothelioma, which suggests that blocking these signal pathways is a therapeutic strategy. Mesothelioma often up-regulated expression of vascular endothelial growth factor (VEGF), epidermal growth factor (EGF) and the corresponding receptors (VEGFR, EGFR) (Strizzi et al. 2001; Lee et al. 2007). Furthermore, an expression level of hepatocyte growth factor (HGF) and the receptor, c-Met, is also augmented in a majority of mesothelioma specimens (Jagadeeswaran et al. 2006; Lee et al. 2015). Molecular targeting agents that inhibit EGF/EGFR or VEGF/VEGFR pathway such as erlotinib, gefinitib and bevacizumab however failed to produce clinical benefits (Govindan et al. 2005; Garland et al. 2007; Dowell et al. 2012; Ceresoli et al. 2013), but an inhibitor to block the HGF/c-Met pathway has not yet been examined for the efficacy to mesothelioma. Previous studies showed that the HGF/c-Met pathway played a crucial role in tumor invasion and metastasis, and blocking the pathway suppressed tumor infiltration into neighboring tissues (Matsumoto and Nakamura 2003). HGF is secreted as a precursor form and the pre-proHGF is subsequently cleaved into α and β chains with elastase actions, which results in a heterodimeric structure of mature HGF protein. The α chain is responsible for the binding to the c-Met receptor and the NK4 is a kind of an internal fragment of HGF, being identical to the α chain sequences with a deletion of 16 amino acids. The NK4 molecules bind to the receptor but do not activate the downstream signal, demonstrating that NK4 is a competitive antagonist to block biological activities of HGF. Further investigations revealed that NK4 also suppressed angiogenesis irrelevant to the VEGF/VEGF pathway and had a potential anti-cancer action by modifying a malignant behavior of tumor cells (Kuba et al. 2000; Sakai et al. 2009). A number of preclinical studies demonstrated that adenoviruses (Ad) expressing the *NK4* gene (Ad-NK4) produced anti-tumor effects on many types of tumors including mesothelioma (Saimura et al. 2002; Murakami et al. 2005; Suzuki et al. 2010). Transduction of mesothelioma with Ad-NK4 inhibited HGF-mediated phosphorylation of c-Met and the cell migration. Injection of Ad-NK4 into subcutaneous mesothelioma retarded the subsequent tumor growth. Moreover, administration of Ad-NK4 did not produce any major adverse effects in vivo (Kishi et al. 2009), and a majority of mesothelioma do not have any genetic mutations at the c-Met locus that produce gain-of-functions (Lee et al. 2015). These preclinical studies and analyses prompted us to conduct a clinical trial at Chiba University Hospital, Chiba, Japan, for chemotherapy-failed mesothelioma patients to examine safety and efficacy of an intrapleural injection of Ad-NK4.

### Previous gene therapy for malignant pleural mesothelioma

Several clinical trials of gene therapy for mesothelioma have been conducted with the *herpes simplex*-*thymidine kinase* (*HSV*-*TK*) gene, or *interferon* (*IFN*)-*α* or -*β* gene at University of Pennsylvania (Sterman et al. 1998, 2005, 2007, 2010, 2011). Some clinical case studies with replication-competent Ad or Ad expressing the *p53* gene also included mesothelioma patients but the detailed information about the mesothelioma cases was unavailable (Liu et al. 2006; Cerullo et al. 2010). The phase I studies with intrapleural administration of the Ad vectors demonstrated that a maximum tolerance dose in the HSV-TK study was up to 5 × 10^13^ virus particles (vp) (Sterman et al. 2005) and that in the IFN-β study was 1 × 10^12^ vp (Sterman et al. 2007). The studies also showed that the Ad vectors induced gene transduction in mesothelioma and did not produce any major adverse effects (Sterman et al. 2005, 2007). These data collectively suggest a feasible clinical study for malignant pleural mesothelioma with an intrapleural injection of Ad-NK4.

## Methods/design

### Study drug

The agent, Ad-NK4, is type 5 Ad containing the expression cassette of the cytomegalovirus promoter-linked a full-length of NK4 cDNA followed by the SV40 T antigen-derived poly A additional signal. The cGMP-grade vector was produced at GMJ Inc, Kobe, Japan, with PER.C6 cells which significantly inhibited generation of replication-competent Ad (RCA) (Fallaux et al. 1998). Ad-NK4 for the clinical study were produced from the master virus bank and each vial contains 1.52 × 10^12^ vp/ml. The Ad-NK4 products were confirmed to be free from various types of microorganisms and endotoxins. We also examined the frequency of RCA produced from the clinical grade vectors with A549 cells and the spike method (Ishii-Watabe et al. 2003). The RCA frequency was less than 1 out of 3 × 10^10^ vp at a sensitivity level that detected 1 RCA out of more than 3 × 10^9^ viruses. The vials were kept in a refrigerator at −80° and the viral titers remained unchanged for more than 5 years under the condition.

### Study design and objectives

**Figure 1:**
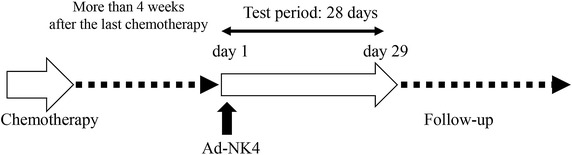
A diagram of the study design. Enrollment of a patient needs more than 4 weeks after the last chemotherapy. Ad-NK4 is injected into the pleural cavity and then the patient will be monitored. The clinical study ends after day 29 but we will follow-up the patient thereafter.

The study design is to administer 100 ml saline solution containing Ad-NK4 into the pleural cavity of mesothelioma patients who are not suitable for a surgical operation and fail to respond to the first-line chemotherapeutic agents (Figure 1). The study includes a dose escalation schedule with Ad-NK4 at 1 × 10^10^, 1 × 10^11^ or 1 × 10^12^ vp per person and a single injection for each dose group consisting of 3 patients. The observation period of the study is 28 days but we follow-up them at an outpatient clinic. The primary endpoints are to investigate safety levels of Ad-NK4 injected into the pleural cavity and adverse effects produced, and to define the maximum tolerance dose. The secondary endpoints are evaluation of anti-tumor effects based on radiological imaging and of any improvements regarding patients’ quality of life and the performance status.

### Subject selection and withdrawal

#### Inclusion criteria

Patients eligible for the study are those who are pathologically diagnosed as malignant pleural mesothelioma of locally advanced or recurrent cases, and not suitable for surgical resection. Those refuse to take the surgery irrespective of their clinical stages are also eligible. In addition, the patients who become refractory to chemotherapy or decline to receive chemotherapeutic agents are eligible. More than 4 weeks are required to be enrolled in the study after completing the precedent chemotherapy. Moreover, they should not have any treatment history of radiotherapy since radiation may produce delayed anti-tumor effects. Patients, aged between 20 and 80 years old, must be fully explained about the study using a document and give written informed consent.

Patients must have pleural effusion to have an enough space in the pleural cavity where Ad-NK4 is injected. The Eastern Co-operative Oncology Performance status must be 0 or 1. Female patients should not be pregnant or be lactating to nurse a baby. Patients must agree to use a physical method of contraception. Life expectancy of the patients will be longer than 3 months. Patients need to have adequate physiological functions in major organs and the laboratory findings must be as follows: white blood cell, ≥3,000/mm^3^ or neutrophil, ≥2,000/mm^3^; platelet, ≥1 × 10^5^/mm^3^; hemoglobin, ≥9.0/dl; total bilirubin, less than 1.5 times of a upper-limit of a normal range of the hospital; aspartate transaminase (AST) and alanine aminotransferase (ALT), less than 2 times of a upper-limit of a normal range of the hospital; alkaline phosphatase, less than 5 times of a upper-limit of a normal range of the hospital; creatinine, <1.5 mg/dl, prothrombin time and activated partial thromboplastin time; within normal range; SPO_2_ (breathing in room air), ≥92%; electric cardiogram, within normal range.

The principle investigator and the medial collaborators including those responsible for monitoring the current study must approve that the patient is in an adequate condition to be enrolled in the study.

#### Exclusion criteria

Patients are not eligible for the study when they have active and uncontrollable infectious diseases, or any serious complications. Patients with a different type of malignancy, either synchronous or metachronous, are not eligible unless they are completely cured or their progression free interval is longer than 2 years. Patients who have symptomatic brain metastatic foci or those who require a treatment for the brain metastasis are excluded. Patients who do not have an enough intra-thoracic space for the viral injection or have participated in other clinical trial(s) with approved or unapproved medicine within 4 weeks before the entry of this study are not allowed to be enrolled. Those who are scheduled to receive another anti-cancer drug(s) during the study period or have received pleurodesis as a mesothelioma treatment are excluded. Patients who have peripheral nerve palsy at above grade 2 level (CTCAE ver 4.0) at the entry or have interstitial diseases and pulmonary fibrosis judged by chest X-ray are not eligible.

Those who have factors that prevent good compliance with the study protocol and the follow-up schedules, including some psychiatric, psychological, familial, social or geographical issues, are regarded as not being suitable for the study. Patients who have already undergone a treatment using Ad vectors or a treatment history of auto- or allograft organs transplantation, those who are positive for HIV antigen, HBV antigen, HCV antibody or HTLV-1 antibody, and those who are judged as inappropriate to participate in the study by the principal investigator and the collaborators are excluded.

### Safety tests in preclinical study

Many preclinical and clinical studies demonstrated safety and biodistributions of Ad that were administered subcutaneously or intravenously (Reynolds et al. 1999; Hackett et al. 2000). In contrast, biodistribution of Ad after an intrapleural injection has not yet investigated with an animal model although a clinical study for mesothelioma reported Ad in the sera after intrapleural administration (Sterman et al. 2007). The clinical trial showed that sera of the patients became negative within a week when they received up to 3 × 10^12^ vp in the thoracic cavity, but no further data in other tissues were included. We thereby conducted a preclinical study to examine biodistribution and possible adverse reactions with mice that received Ad-NK4 in the pleural cavity.

#### Acute toxicity in intrapleural injection

We injected Ad-NK4 at 1.52 × 10^11^ vp/mouse (0.1 ml) or phosphate-buffered saline (PBS, 0.1 ml) as a control into the pleural cavity of CrlJ:CD1 mice (Japan Charles River, Yokohama, Japan). The maximum human dose used in the clinical study is 2 × 10^10^ vp/kg when an average body weight of patients is 50 kg, and a dose for the safety test with mice is 7.6 × 10^12^ vp/kg when that of mice is 20 g. The administered dose in mice thereby corresponded to 380 times of the maximum human dose in the clinical study. We examined changes of body weights, biochemical tests, viral DNA distributions, transgene expressions and pathological findings until day 14 as the acute toxicity.

**Figure 2:**
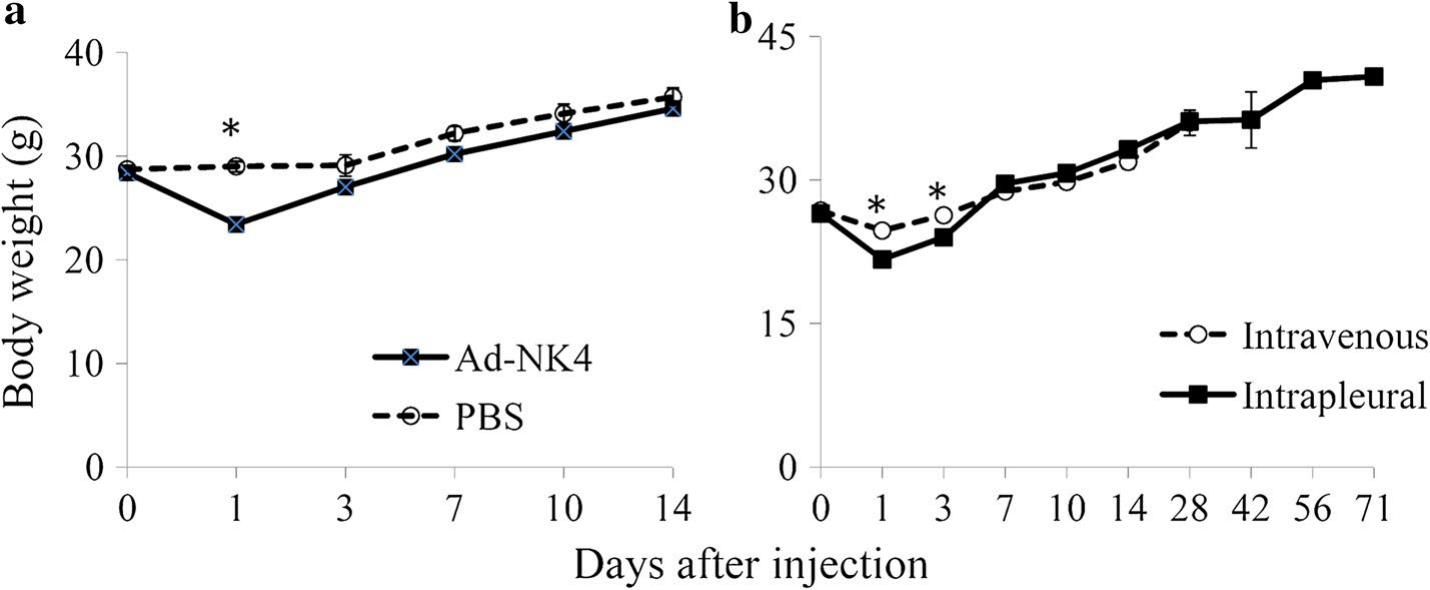
Body weight changes in mice. **a** Ad-NK4 or PBS was administered in the pleural cavity of CrlJ:CD1 mice (n = 6). **b** Ad-NK4 were administered intravenously or into the pleural cavity of CrlJ:CD1 mice (n = 5). Average and SEs are shown. *P < 0.01 (analyzed with one-way analysis of variance).

*Body weight* We measured a body weight as a marker for general mouse condition (Figure 2a). The body weight of mice inoculated with Ad-NK4 temporally decreased on day 1 after the injection but the increase rate was not different from that of mice injected with PBS thereafter. Ad-NK4 administration may transiently suppress water or food intake in mice but produce little effects after day 2.

*Hematological and biochemical data* We conducted complete blood counting and biochemical analyses of the mice which received Ad-NK4 or PBS (Tables 1, 2). Numbers of white blood cells (WBC) and platelets were relatively higher in mice injected with Ad-NK4 than those with PBS (WBC day 14, P = 0.10; platelet day 1, P = 0.08) but the results of other laboratory tests were not markedly different between the groups (Table 1). The average blood glucose value was lower in Ad-NK4-injected than in PBS-injected mice on day 1 (P < 0.01), which could be associated with the temporal body weight loss on day 1. AST and ALT values in Ad-NK4-injected mice was greater than those in the control mice on days 1 and 7 (P < 0.05) but the elevation became statistically insignificant thereafter (day 14: AST, P = 0.055; ALT, P = 0.089). Interestingly, the alkaline phosphatase values were lower in Ad-NK4-injected mice than in control mice on day 1 (P < 0.05) but was not different thereafter. Moreover, the lactate dehydrogenase values were not statistically different between the groups through days 1–14. These data suggested that intrapleural injection of Ad-NK4 produced liver damages rather than cholestatic injury, and the liver dysfunction came to be normalized thereafter. Differential data regarding the liver damages may be attributable to the fact that Ad vectors are preferentially integrated into Kupffer cells rather than hepatocytes (Lieber et al. 1997). A few mice showed elevation of creatine phosphokinase values in both groups, the control group on days 7 and 14 and Ad-NK4-injected group on day 14, which could be due to some tissue damages at blood samplings.

**Table 1:** Complete blood counting data of mice injected with Ad-NK4 or PBS

Test	Day 1		Day 7		Day 14	
	Ad-NK4	PBS	Ad-NK4	PBS	Ad-NK4	PBS
WBC (×10^2^/μl)	17 ± 2	23 ± 4	36 ± 10	20 ± 2	83 ± 26	27 ± 7
RBC (×10^4^/μl)	748 ± 20	674 ± 54	652 ± 32	736 ± 11	734 ± 32	713 ± 58
Hgb (g/dl)	13.3 ± 0.4	11.7 ± 0.7	11.2 ± 0.6	12.4 ± 0.1	12.6 ± 0.6	12.5 ± 0.7
Hct (%)	44.7 ± 1.2	40.2 ± 3.1	36.8 ± 2.0	40.8 ± 0.5	38.7 ± 1.8	37.8 ± 3.0
MCV (fl)	59.8 ± 0.4	59.7 ± 1.0	56.4 ± 1.1	55.4 ± 0.6	52.7 ± 0.1	53.0 ± 0.4
MCH (pg)	17.7 ± 0.2	17.4 ± 0.4	17.2 ± 0.4	16.9 ± 0.3	17.1 ± 0.1	17.7 ± 0.6
MCHC (%)	29.6 ± 0.2	29.1 ± 0.7	30.4 ± 0.4	30.4 ± 0.4	32.5 ± 0.1	33.3 ± 1.0
Plt (×10^4^/μl)	109.9 ± 12.0	67.9 ± 18.8	55.4 ± 14.6	49.3 ± 10.6	83.9 ± 34.4	48.2 ± 23.8

Data show average and SE values (n = 6). CrlJ:CD1 mice injected with Ad-NK4 were 5-week old male. Data of untreated CrlJ:CD1 male mice at 11-week old (Japan Charles river, Japan) as follows.

WBC, 20 ± 6.9 × 10^2^/μl; RBC, 943 ± 61 × 10^4^/μl; Hgb, 14.8 ± 1.0 g/dl; Hct, 45.3 ± 2.4%; MCV, 48.1 ± 1.9 fl; MCH, 15.7 ± 0.5 pg; MCHC, 32.6 ± 0.5%; Plt, 144.4 ± 18.9 × 10^4^/μl.

**Table 2:** Biochemical data of mice injected with Ad-NK4 or PBS

Test	Day 1		Day 7		Day 14	
	Ad-NK4	PBS	Ad-NK4	PBS	Ad-NK4	PBS
TP (g/dl)	5.4 ± 0.2	4.7 ± 0.1	4.6 ± 0.1	4.9 ± 0.1	5.0 ± 0.2	4.8 ± 0.1
ALB (g/dl)	2.8 ± 0.0	2.8 ± 0.1	2.6 ± 0.0	3.0 ± 0.0	2.8 ± 0.1	2.8 ± 0.0
BUN (mg/dl)	25.8 ± 1.0	23.6 ± 1.4	22.8 ± 0.9	24.5 ± 1.0	25.2 ± 0.9	28.0 ± 1.6
CRE (mg/dl)	0.14 ± 0.01	0.16 ± 0.01	0.13 ± 0.01	0.15 ± 0.02	0.14 ± 0.02	0.15 ± 0.01
Na (mEq/l)	153 ± 1	149 ± 1	151 ± 1	151 ± 1	149 ± 1	151 ± 1
K (mEq/l)	4.8 ± 0.2	4.4 ± 0.2	4.1 ± 0.2	3.9 ± 0.4	5.5 ± 0.5	4.3 ± 0.1
Cl (mEq/l)	101 ± 2	104 ± 0	106 ± 1	104 ± 1	105 ± 1	103 ± 2
Ca (mg/dl)	9.2 ± 0.1	9.6 ± 0.1	9.5 ± 0.1	9.5 ± 0.0	9.3 ± 0.1	9.5 ± 0.2
IP (mg/dl)	7.9 ± 0.4	9.7 ± 0.9	8.1 ± 0.1	7.8 ± 0.3	10.0 ± 0.9	7.1 ± 0.4
AST (IU/l)	191 ± 18	80 ± 18	383 ± 273	93 ± 32	347 ± 96	95 ± 13
ALT (IU/l)	131 ± 30	26 ± 7	312 ± 238	28 ± 3	244 ± 96	29 ± 3
ALP (IU/l)	198 ± 10	378 ± 43	407 ± 60	408 ± 79	448 ± 62	324 ± 30
LDH (IU/l)	3,352 ± 359	2,686 ± 826	2,535 ± 847	2,142 ± 287	2,622 ± 592	2,087 ± 271
CPK (IU/l)	1,262 ± 408	1,032 ± 412	432 ± 70	3,734 ± 3,283	4,493 ± 3,156	1,297 ± 387
T-CHO (mg/dl)	219 ± 11	147 ± 5	161 ± 8	169 ± 8	153 ± 8	176 ± 7
TG (mg/dl)	131 ± 17	109 ± 3	74 ± 13	40 ± 7	57 ± 6	119 ± 27
T-BIL (mg/dl)	0.05 ± 0.01	0.04 ± 0.00	0.09 ± 0.01	0.10 ± 0.01	0.08 ± 0.01	0.07 ± 0.01
GLU (mg/dl)	106 ± 7	169 ± 3	187 ± 12	173 ± 3	197 ± 8	192 ± 15

Data show average and SE values (n = 6). CrlJ:CD1 mice injected with Ad-NK4 were 5-week old male. Data of untreated CrlJ:CD1 male mice at 11-week old (Japan Charles river, Japan) as follows.

TP, 4.2 ± 0.3 g/dl; ALB, 2.3 ± 0.3 g/dl; BUN, 33.8 ± 8.6 mg/dl; CRE, 0.3 ± 0.09 mg/dl; Na, 153 ± 2 mEq/l; K, 4.6 ± 0.4 mEq/l; Cl, 106.7 ± 4.3 mEq/l; Ca, 8.8 ± 0.3 mg/dl; IP, 7.9 ± 1.5 mg/dl; AST, 76 ± 11 IU/l; ALT, 32 ± 7 IU/l; ALP, 82 ± 31 IU/l; LDH, not available; CPK, 249 ± 96 IU/l; T-CHO, 151 ± 44 mg/dl; TG, 78 ± 58 mg/dl; T-BIL, 0.08 ± 0.03 mg/dl; GLU, 101 ± 35 mg/dl.

*Biodistribution of Ad-NK4 DNA* We examined tissue distributions of Ad-NK4 DNA with the real time polymerase chain reaction technique and calculated the copy numbers per 25 ng of genomic DNA extracted from respective tissues (Table 3). The DNA was detected in all the tissues tested, and the copy numbers in tissues were maximal on day 1 and decreased thereafter. Lung showed the largest amounts of DNA integration, and the copy numbers in liver were stable for 2 weeks after the injection in contrast to those in other tissues which decreased the numbers subsequently. These data suggested that Ad-NK4 administered in the pleural cavity infected lung through mesothelium and were transferred into systemic circulation. The copy numbers distributed in whole body became low thereafter but liver continuously harbored Ad-NK4.

**Table 3:** Biodistribution of Ad-NK4 in mice injected into the pleural cavity

Tissues	Copy numbers of Ad-NK4 per 25 ng of total genomic DNA		
	Day 1	Day 7	Day 14
Brain	3.9 × 10^5^ ± 1.7 × 10^5^	3.4 × 10^3^ ± 1.7 × 10^3^	2.3 × 10^2^ ± 1.0 × 10^2^
Lung	3.1 × 10^8^ ± 2.4 × 10^8^	1.3 × 10^5^ ± 3.9 × 10^4^	7.5 × 10^3^ ± 3.9 × 10^3^
Stomach	1.5 × 10^6^ ± 1.1 × 10^6^	1.9 × 10^3^ ± 4.3 × 10^2^	3.4 × 10^2^ ± 1.4 × 10^2^
Spleen	6.4 × 10^5^ ± 5.2 × 10^5^	4.2 × 10^4^ ± 1.6 × 10^4^	2.6 × 10^3^ ± 1.5 × 10^3^
Kidney	1.3 × 10^4^ ± 4.3 × 10^3^	2.0 × 10^3^ ± 6.3 × 10^2^	2.1 × 10^3^ ± 9.6 × 10^2^
Liver	3.2 × 10^4^ ± 1.9 × 10^4^	1.9 × 10^4^ ± 8.2 × 10^3^	1.2 × 10^4^ ± 1.1 × 10^4^
Small intestine	1.1 × 10^4^ ± 3.2 × 10^3^	3.9 × 10^2^ ± 2.0 × 10^2^	6.5 × 10^2^ ± 3.7 × 10^2^
Colon	1.3 × 10^4^ ± 7.1 × 10^3^	9.8 × 10^2^ ± 8.9 × 10^2^	1.0 × 10^2^ ± 35
Testis	4.5 × 10^3^ ± 2.1 × 10^3^	2.3 × 10^2^ ± 94	45 ± 24
Blood	1.2 × 10^4^ ± 4.6 × 10^3^	2.4 × 10^3^ ± 1.5 × 10^3^	2.0 × 10^2^ ± 92

Data show average and SE values (n = 6). DNA from respective tissues was amplified to detect a Ad E4 region with the real time polymerase chain reactions as follows, a forward primer, 5′-CACCACCTCCCGGTACCATA-3′, a reverse primer, 5′-CCGCACCTGGTTTTGCTT-3′, and 40 cycles at 62°C for annealing. Ad reference material (VR-1516, ATCC, Manassas, VA, USA) was used as a control.

*Expression of NK4 mRNA* We also examined transcript levels of NK4 in tissues of mice injected with Ad-NK4 in the pleural cavity (Table 4). The expression was significant high in lung on day 1 and subsequently decreased. The rapid reduction was also observed in other tissues such as stomach. In contrast, the expression in liver was relatively stable.

**Table 4:** Expression of the NK4 gene in mice injected with Ad-NK4 into the pleural cavity

Tissues	Relative NK4 mRNA levels per 100 ng of synthesized cDNA		
	Day 1	Day 7	Day 14
Brain	41.4 ± 8.0	2.4 ± 0.6	1.2 ± 0.3
Lung	21,563.1 ± 7,314.8	94.8 ± 37.9	10.5 ± 4.8
Stomach	244.9 ± 120.4	1.4 ± 0.4	6.2 ± 2.3
Spleen	111.6 ± 63.2	9.9 ± 2.9	3.6 ± 1.5
Kidney	13.7 ± 5.3	4.5 ± 2.4	1.2 ± 0.3
Liver	28.6 ± 2.8	25.3 ± 16.1	16.5 ± 5.4
Small intestine	34.6 ± 9.4	31.2 ± 2.0	9.3 ± 1.3
Colon	4.8 ± 1.5	1.7 ± 0.4	1.7 ± 0.4
Testis	2.8 ± 0.6	1.8 ± 0.2	0.5 ± 0.1

Data show average and SE values (n = 6). First-strand cDNA was amplified with the real time polymerase chain reaction and with the NK4 primers, forward 5′-CGAGGCCATGGTGCTATACTC-3′, and reverse 5′-TCAGCGCATGTTTTAATTGCA-3′, and the glyceraldehyde 3-phosphate dehydrogenase primers, forward 5′-AGCAAGGACACTGAGCAAGAG-3′, and reverse 5′-TATTATGGGGGTCTGGATG-3′, at 62°C for annealing and for 40 cycles. The NK4 mRNA amounts were estimated with the glyceraldehyde 3-phosphate dehydrogenase as a control and expressed as an arbitrary unit based on a standard sample.

**Figure 3:**
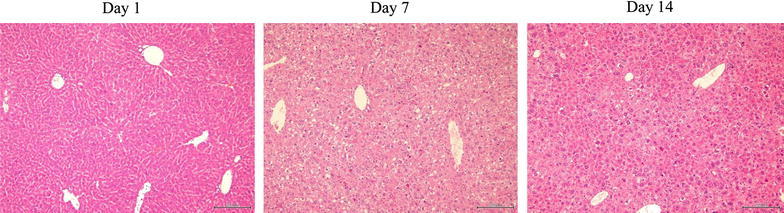
Representative liver sections of mice injected with Ad-NK4 on days 1, 7 and 14. The sections were stain with hematoxylin–eosin and the *bars* indicate 200 μm.

**Figure 4:**
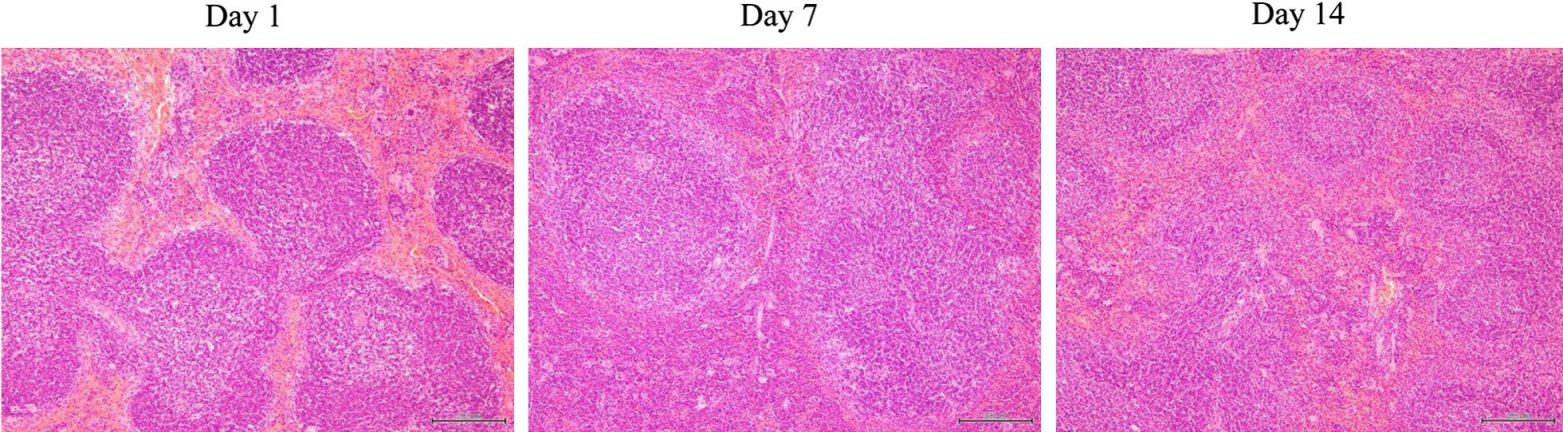
Representative spleen sections of mice injected with Ad-NK4 on days 1, 7 and 14. The sections were stain with hematoxylin–eosin and the *bars* indicate 200 μm.

*Pathological findings* Ad-NK4 injected into the pleural cavity induced mild degeneration of hepatocytes and infiltration of inflammatory cells in liver, but not necrosis on day 7 (Figure 3). The liver damages became less significant on day 14. Spleen showed mildly enlarged follicles in the lymph nodes but the findings became undetectable on day 14 (Figure 4). Other tissues including brain, lung, stomach, kidney, intestine, colon and testis did not show any pathological changes.

#### Long-term effects in intrapleural injection

We examined long-term effects caused by Ad-NK4 with two different administration routes, intravenous and intrapleural injections. The body weight of mice received Ad-NK4 in the intrapleural cavity was less than that of mice injected intravenously on days 1 and 3 (P < 0.01), but was not different thereafter (Figure 2b). Biochemical analyses on liver functions showed that AST and ALT values in the case of intravenous injections peaked on day 7 and then decreased thereafter (Table 5). In contrast, changes of these data in the intrapleural injection were retarded and less significant in comparison with those in the intravenous injection (Tables 2, 5). Nevertheless, the transaminase values in the intrapleural injection were still above the normal range even on day 71. These data suggest that intrapleural injection of Ad vectors induced prolonged liver dysfunctions. We also compared tissue distributions of Ad-NK4 with two different administration routes (Table 6). An intravenous injection of Ad-NK4 caused wide spread of Ad DNA in all the tissues tested, and liver was the major organ that harbored the DNA on day 28. The copy numbers of Ad vectors in these tissues peaked on day 1 and decreased thereafter, but parenchymal organs showed constant Ad integrations until day 28. Tissue distributions and the kinetics of Ad-NK4 DNA after the intrapleural injection were similar to and less than those after the intravenous administration. The copy numbers in the parenchymal organs remained similar from days 14 to 71 in the intrapleural injection. These data indicated that Ad vectors integrated in the parenchymal organs were not eliminated even 10 weeks after the intrapleural injection, which may be linked with turnover levels of cells in these organs.

**Table 5:** Comparison of AST and ALT values in different administration routes

Administration	Enzyme activity (IU/L)						
	Day 1	Day 7	Day 14	Day 28	Day 42	Day 56	Day 71
AST							
Intravenous	801 ± 255	1,390 ± 492	644 ± 200	281 ± 81			
Intrapleural			214 ± 65	219 ± 38	202 ± 30	173 ± 23	169 ± 31
ALT							
Intravenous	356 ± 112	1,881 ± 648	1,031 ± 320	223 ± 62			
Intrapleural			361 ± 172	180 ± 63	205 ± 55	76 ± 12	65 ± 14

Data show average and SE values (n = 5). CrlJ:CD1 mice injected with Ad-NK4 were 5-week old male. Data of untreated CrlJ:CD1 male mice at 11-week old (Japan Charles river, Japan) as follows.

AST, 76 ± 11 IU/l; ALT, 32 ± 7 IU/l.

**Table 6:** Biodistribution of Ad-NK4 in different administration routes

Tissues	Copy numbers of Ad-NK4 per 25 ng of total genomic DNA				
Route	Day 1	Day 7	Day14	Day 28	Day 71
Brain					
Intravenous	6.5 × 10^5^ ± 6.0 × 10^5^	3100 ± 2200	520 ± 280	2500 ± 690	
Intraplueral			180 ± 80	370 ± 130	140 ± 50
Lung					
Intravenous	1.9 × 10^6^ ± 8.9 × 10^5^	3.3 × 10^5^ ± 1.2 × 10^5^	9.9 × 10^4^ ± 4.0 × 10^4^	1.3 × 10^5^ ± 6.8 × 10^4^	
Intrapleural			1.9 × 10^4 ^± 4.1 × 10^3^	7200 ± 2600	2.1 × 10^4^ ± 7600
Spleen					
Intravenous	1.3 × 10^6^ ± 1.3 × 10^6^	6.2 × 10^4 ^± 2.3 × 10^4^	1.7 × 10^5^ ± 3.9 × 10^4^	2.0 × 10^5^ ± 7.9 × 10^4^	
Intrapleural			5.3 × 10^4^ ± 2.2 × 10^4^	7500 ± 2500	4.0 × 10^4 ^± 1.3 × 10^4^
Liver					
Intravenous	5.1 × 10^7^ ± 4.0 × 10^7^	9.5 × 10^5^ ± 3.5 × 10^5^	8.8 × 10^5^ ± 1.6 × 10^5^	8.7 × 10^5^ ± 3.7 × 10^5^	
Intrapleural			2.3 × 10^4^ ± 1.6 × 10^4^	1.4 × 10^4^ ± 6900	5000 ± 1700
Kidney					
Intravenous	1.2 × 10^6^ ± 2.6 × 10^5^	2.0 × 10^4^ ± 1.2 × 10^4^	2.7 × 10^4 ^± 1.6 × 10^4^	2.8 × 10^4 ^± 9200	
Intrapleural			2700 ± 660	1.0 × 10^3 ^± 2000	1.7 × 10^3 ^± 3600
Testis					
Intravenous	1.1 × 10^5^ ± 4 .8 × 10^4^	190 ± 170	70 ± 30	7,200 ± 7,000	
Intrapleural			120 ± 20	40 ± 20	440 ± 190

Data show average and SE values (n = 5). Blood was negative for Ad-NK4 at any time irrespective of injection routes. The procedure to detect the copy numbers is the same as that in Table 3.

## Discussion

There are several points to investigate in the current clinical research, which includes antibody production, blocking of the HGF/c-Met signaling and clinical outcomes. Previous studies showed that almost all the patients developed neutralizing antibody against Ad structural proteins within 1 week after the intrapleural injection, and that the antibody production in an intrapleural administration seemed to have a similar kinetics as that in an intravenous injection (Molnar-Kimber et al. 1998). Moreover, the antibody level in the pleural effusion was the same as that in the serum (Molnar-Kimber et al. 1998). Peripheral T cells proliferated after a single Ad injection (Molnar-Kimber et al. 1998), suggesting that T cells in general populations were already primed with Ad probably due to their previous respiratory infections. Likewise, humoral responses against Ad protein can be secondary immune responses. Interestingly, antibody was generated even against the transgene (Sterman et al. 2007). These immune responses did not induce serious adverse reactions, and pre-existing humoral and cellular immunity prior to the Ad administration did not inhibit subsequent Ad-mediated gene transduction. Sterman et al. (2010) examined whether multiple Ad administrations in a short period could increase gene transduction level and showed that the second injection 3 days after the first Ad administration increased the transgene expression probably because neutralizing anti-Ad antibody was not yet produced on day 3. We will therefore examine antibody titers against Ad hexon protein in patients’ serum and pleural effusion in the present study.

We also plan to conduct quantitative analyses of NK4 molecules with an enzyme-linked immunosorbent assay system and further examine inhibition of the HGF/c-Met signaling by Ad-derived NK4. A pleural effusion of the patient injected with Ad-NK4 is tested for the ability to dephosphorylate c-Met molecules in human mesothelioma cell lines or in patients-derived tumor cells if the samples are available. It is uncertain at this moment whether anti-NK4 antibody is produced by Ad-NK4 administration since NK4 protein is naturally detected in human as a cleaved form of HGF. We speculate that NK4 molecules are less likely to be recognized as foreign substance by a host immune system and antibody against NK4 may not be produced. The minimal concentration of NK4 molecules to inhibit growth of mesothelioma in vivo is currently unknown. Clinical outcomes by Ad-NK4 administration will be influenced by dependence levels of mesothelioma on the HGF/c-Met pathway for the cell growth and on the angiogenesis which is produced by interactions between the tumors and the surrounding interstitial tissues. Previous studies showed that the transgene expression was detected only at superficial cell layers of the tumor tissues (Sterman et al. 2005) and we presume that changing body position after Ad injection can be beneficial to augment efficacy of the infections. Moreover, NK4 molecules are secretary protein and are consequently accumulated in the pleural space. Stability of NK4 molecules in vivo is however not well understood and sequential quantification of NK4 protein in the pleural effusion is important to estimate the NK4-mediated inhibition of HFG/c-Met pathways.

Previous clinical studies indicated that overall clinical responses were greater than the estimation based on the gene transduction efficacy (Sterman et al. 2005, 2007). Moreover, tumor regression detected by tumor imaging techniques became evident even a few months after the Ad administration (Sterman et al. 2005, 2007), suggesting that the anti-tumor effects were attributable to possible systemic immune responses. In addition, post-treatment patients’ sera reacted with molecules that were expressed in several human mesothelioma cell lines, whereas pre-treatment sera of the same patients did not. The molecular sizes that the antibody reacted were different among the respective cases, but the data suggested possible shared tumor antigens among human mesothelioma. Generation of antibody against unidentified molecules does not directly indicate activation of cell-mediated immunity against the putative antigen(s), but helper T cells, involved in either humoral or cellular immune responses, are activated by the same major histocompatibility complex class II-binding peptide(s). Production of antibody against putative tumor antigen molecules thereby suggests induction of cytotoxic T cells targeting the molecules. Cell death caused by Ad vectors can facilitate uptake of putative tumor antigens by professional antigen presenting cells and Ad vectors can work as an adjuvant together with cytokines released from recruiting inflammatory cells. These immune responses in the previous studies did not induce serious adverse reactions, and pre-existing humoral and cellular immunity prior to the Ad administration did not inhibit subsequent Ad-mediated gene transduction (Molnar-Kimber et al. 1998; Sterman et al. 2005, 2007). Migration of Ad vectors from an intrapleural space into systemic circulation raises an issue of shedding from the patient body. We will monitor presence of the Ad vectors with the polymerase chain reaction in blood, urine and sputum of the tested individuals. The precedent study showed that sera of the patients were positive for Ad vectors for a week after an intrapleural injection (Sterman et al. 2007). It is however currently unknown how immune responses play a role in the decrease of Ad DNA from the systemic circulation.

Mesothelioma is histologically classified into three subtypes, epithelioid, biphasic and sarcomatoid types. The sensitivity to chemotherapy is different among the subtypes and prognosis of sarcomatoid type is in general the worst. A recent study also indicated a polyclonal origin of mesothelioma, which subsequently influences the chemosensitivity and the prognosis depending on a ratio of heterogeneity within tumors (Comertpay et al. 2014). A clinical response to Ad-NK4 can also be varied among the histological subtypes as well. The present study however does not take the subtype into consideration at the patient enrollment since the study is a first-in-human trial regarding NK4 molecules and the primary endpoint is to investigate the safety of Ad-NK4. An examination of possible anti-tumor effects is the secondary endpoint but the patient number is too small to conclude the clinical outcomes.

The present protocol is to evaluate safety and then efficacy of Ad-NK4 administered in the pleural cavity. Confirmation of safety of the gene therapy will promote next possible studies which include a combination of Ad-NK4 and the first-line chemotherapy to a chemotherapy-naïve patient, and a phase II study with a maximum-tolerance dose of Ad-NK4.

## Abbreviations

Ad: adenoviruses
ALT: alanine aminotransferase
AST: aspartate transaminase
CDDP: cisplatin
EGF: epidermal growth factor
EGFR: epidermal growth factor receptor
HGF: hepatocyte growth factor
HSV-TK: herpes simplex-thymidine kinase
IFN: interferon
PBS: phosphate-buffered saline
PEM: pemetrexed
RCA: replication-competent adenoviruses
VEGF: vascular endothelial growth factor
VEGFR: vascular endothelial growth factor receptor
Vp: virus particles
WBC: white blood cells
